# Supplementary material for: Event dependent overall survival in the population-based LIFE-Adult-Study
Source: PLoS One. 2022 Dec 1;17(12):e0278069. doi: 10.1371/journal.pone.0278069 (PMC9714713; doi:10.1371/journal.pone.0278069)
Supplement: Attachment — Submitted filename: Response to Reviewers.docx [file pone.0278069.s001.docx]

PONE-D-22-10390

Dear PhD Dr. Zimmerman,

Thank you for giving us the opportunity to submit a revised draft of the manuscript entitled “Event dependent overall survival in the population-based LIFE-Adult-Study” by Zeynalova, Rillich et al. for publication in your Journal PLOS ONE. We appreciate the time and effort that you and the reviewers dedicated to providing feedback on our manuscript and are grateful for the comments on and valuable improvements to our paper.

We have incorporated the suggestions made by the reviewers. Those changes are highlighted within the manuscript. Please see below, for a point-by-point response to the reviewers’ comments and concerns.

With best regards,

Samira Zeynalova and Katja Rillich

Dear PhD Dr. Zimmerman,

We thank you for recognising the value of our report and will consider all suggestions.

PONE-D-22-10390

Event dependent overall survival in the population-based LIFE-Adult-Study

PLOS ONE

Dear Dr. Rillich,

Thank you for submitting your manuscript to PLOS ONE. After careful consideration, we feel that it has merit but does not fully meet PLOS ONE’s publication criteria as it currently stands. Therefore, we invite you to submit a revised version of the manuscript that addresses the points raised during the review process.

Please submit your revised manuscript by Sep 23 2022 11:59PM. If you will need more time than this to complete your revisions, please reply to this message or contact the journal office at [plosone@plos.org](mailto:plosone@plos.org). When you're ready to submit your revision, log on to https://www.editorialmanager.com/pone/ and select the 'Submissions Needing Revision' folder to locate your manuscript file.

Please include the following items when submitting your revised manuscript:

- A rebuttal letter that responds to each point raised by the academic editor and reviewer(s). You should upload this letter as a separate file labeled 'Response to Reviewers'. It has been done
- A marked-up copy of your manuscript that highlights changes made to the original version. You should upload this as a separate file labeled 'Revised Manuscript with Track Changes'. It has been done
- An unmarked version of your revised paper without tracked changes. You should upload this as a separate file labeled 'Manuscript'. It has been done

If you would like to make changes to your financial disclosure, please include your updated statement in your cover letter. Guidelines for resubmitting your figure files are available below the reviewer comments at the end of this letter.

If applicable, we recommend that you deposit your laboratory protocols in protocols.io to enhance the reproducibility of your results. Protocols.io assigns your protocol its own identifier (DOI) so that it can be cited independently in the future. For instructions see: <https://journals.plos.org/plosone/s/submission-guidelines#loc-laboratory-protocols>. Additionally, PLOS ONE offers an option for publishing peer-reviewed Lab Protocol articles, which describe protocols hosted on protocols.io. Read more information on sharing protocols at <https://plos.org/protocols?utm_medium=editorial-email&utm_source=authorletters&utm_campaign=protocols>.

We look forward to receiving your revised manuscript.

Journal Requirements:

When submitting your revision, we need you to address these additional requirements.

1. Please ensure that your manuscript meets PLOS ONE's style requirements, including those for file naming. The PLOS ONE style templates can be found at https://journals.plos.org/plosone/s/file?id=wjVg/PLOSOne_formatting_sample_main_body.pdf and 
https://journals.plos.org/plosone/s/file?id=ba62/PLOSOne_formatting_sample_title_authors_affiliations.pdf

We thank the editors and reviewers for this recomendation and will take it into account.

2. PLOS requires an ORCID iD for the corresponding author in Editorial Manager on papers submitted after December 6th, 2016. Please ensure that you have an ORCID iD and that it is validated in Editorial Manager. To do this, go to ‘Update my Information’ (in the upper left-hand corner of the main menu), and click on the Fetch/Validate link next to the ORCID field. This will take you to the ORCID site and allow you to create a new iD or authenticate a pre-existing iD in Editorial Manager. Please see the following video for instructions on linking an ORCID iD to your Editorial Manager account: <https://www.youtube.com/watch?v=_xcclfuvtxQ>

We thank the editors and reviewers for this recomendation. We have taken it into account and have already done everything as suggested. The ORCID iD is: 0000-0002-4734-8376

3. Thank you for stating the following in the Acknowledgments Section of your manuscript:

"Leipzig Research Centre for Civilization Diseases (LIFE) is an organizational unit affiliated to the Medical Faculty of the University Leipzig. LIFE was funded by means of the European Union, by the European Regional Development Fund (ERDF) and by funds of the Free State of Saxony within the framework of the excellence initiative (project numbers 713-241202, 713- 241202, 14505/2470, 14575/2470). The authors thank the participants of the LIFE-Adult-Study for taking part in the study and the complete LIFE-Adult team for organizing the course of the study and performing the examinations. Data evaluation for this publication was supported by Sanofi Genzyme, Germany, by a non-restricted scientific grant. 
We thank Ulrike Schoenwiese und Ute Enders for technical assistance and search in the registry."

We note that you have provided funding information that is not currently declared in your Funding Statement. However, funding information should not appear in the Acknowledgments section or other areas of your manuscript. We will only publish funding information present in the Funding Statement section of the online submission form. 
Please remove any funding-related text from the manuscript and let us know how you would like to update your Funding Statement. Currently, your Funding Statement reads as follows:

"The author(s) received no specific funding for this work."

Please include your amended statements within your cover letter; we will change the online submission form on your behalf.

We thank the editors and reviewers for this comment.

It is the case that the LIFE study was funded, but not this project, so we delete the sentences „LIFE was funded by means of the European Union, by the European Regional Development Fund (ERDF) and by funds of the Free State of Saxony within the framework of the excellence initiative (project numbers 713-241202, 713- 241202, 14505/2470, 14575/2470)“ and „Data evaluation for this publication was supported by Sanofi Genzyme, Germany, by a non-restricted scientific grant „ from the text in manuskript.

4. We note that the grant information you provided in the ‘Funding Information’ and ‘Financial Disclosure’ sections do not match.

When you resubmit, please ensure that you provide the correct grant numbers for the awards you received for your study in the ‘Funding Information’ section.

We thank the editors and reviewers for this comment. But since we have not received any additional grants for this specific work, 'Financial Disclosure' section can remain as it was.

[Note: HTML markup is below. Please do not edit.]

Reviewers' comments:

Reviewer's Responses to Questions

**Comments to the Author**

1. Is the manuscript technically sound, and do the data support the conclusions?

The manuscript must describe a technically sound piece of scientific research with data that supports the conclusions. Experiments must have been conducted rigorously, with appropriate controls, replication, and sample sizes. The conclusions must be drawn appropriately based on the data presented.

Reviewer #1: Yes

Reviewer #2: Yes

2. Has the statistical analysis been performed appropriately and rigorously?

Reviewer #1: Yes

Reviewer #2: Yes

3. Have the authors made all data underlying the findings in their manuscript fully available?

The [PLOS Data policy](http://www.plosone.org/static/policies.action#sharing) requires authors to make all data underlying the findings described in their manuscript fully available without restriction, with rare exception (please refer to the Data Availability Statement in the manuscript PDF file). The data should be provided as part of the manuscript or its supporting information, or deposited to a public repository. For example, in addition to summary statistics, the data points behind means, medians and variance measures should be available. If there are restrictions on publicly sharing data—e.g. participant privacy or use of data from a third party—those must be specified.

 We have tried to answer all the questions below and hope that everything has been thought of.

Reviewer #1: No

Reviewer #2: Yes

4. Is the manuscript presented in an intelligible fashion and written in standard English?

PLOS ONE does not copyedit accepted manuscripts, so the language in submitted articles must be clear, correct, and unambiguous. Any typographical or grammatical errors should be corrected at revision, so please note any specific errors here.

Reviewer #1: Yes

Reviewer #2: Yes

5. Review Comments to the Author

Please use the space provided to explain your answers to the questions above. You may also include additional comments for the author, including concerns about dual publication, research ethics, or publication ethics. (Please upload your review as an attachment if it exceeds 20,000 characters)

Reviewer #1: Dear authors, thank you for giving me the opportunity to review this manuscript. This research has an excellent rigor and provide some essential contributions to the field.

On the other hand the authors should consider some improvements for their review.
Line 112-131 it is not clear, there the information of cause of death came from. Are these diagnosis from the LIFE study, or the information from the death certificates. However the authors should explain if causes of death information is used. Are these underlying causes, any diagnosis or something else?
Otherwise the authors should make some explanations for specific diagnosis. Osteoporosis is not a common underlying cause. It will be used as proxy for specific risks like falls.
Also individuals with osteoporosis generally have higher mortality risk, because it is more likely in age grougs 80 and 90 +. These comparisions are more plausible if comorbidities are considered. Please look at the paper of Ensrud K, Kats A, Boyd C, et al. Association of disease definition, comorbidity burden, and prognosis with hip fracture probability among late-life women in JAMA Intern Med.

Renal insufficieny is also a secondary diagnosis.

Consequently the authors should define between underlying causes/major diagnosis and secondary diagnosis.

Author response: We thank the reviewer for acknowledging the value of our report.

We agree with the reviewer’s comment that the cause of death is unclear.

The causes of death are unknown to us. The study demonstrates only the role of different diseases in the overall survival time. Therefore, our primary endpoint was only overall survival, defined as the time between the date of basisline examination of participant and the date of death as a result of any cause. We have revised and supplemented the text to acknowledge this statement with sentenses “OS was the only endpoint in this work, as the causes-of-death are still unknown or undetermined in the LIFE-Adult-Study.” (Line no. 107-108)

Generally the authors should used a delayed-entry model, while LiFE individuals are entering the study population at different time points. So they have differtent individual and age-specific mortality risk before and after entering LIFE

Author response: We agree with the reviewer’s comment that all LiFE individuals are entering the study at different time points. The overall survival was defined for each participant as the time between the date of basisline examination (of **each** participant) and the date of death as a result of any cause. All living participants were censored at the time of last information from the saxonian population register. In the multivariate Cox regressionmodels for overall survival we adjusted all the diagnoses reported to us with known risk factors as age, gender, BMI, and smoking status.
The authors should also compensate the number of models. For the reader it is not almost comprehensive to interpret the findings from various models and tables.

Author response: We agree with the reviewer’s comment that reader it is not almost comprehensive to interpret the findings from various models and we have renamed our modelst and hope that it is now easier to understand.

175 (old 174) **Univariate analysis (OS according to each comorbidities)**

185 (old 184) **Cox regression model (OS according to each comorbidities adjusted for age and sex)**

194-195 (old 193) **Multivariate Analysis**: **Cox regression model for all reported comorbidities adjusted for age, sex, BMI and smoking status.**

206-207 (old 203) **Optimal Model: Cox regression model for all signifffikant and relevant comorbidities adjusted for age, sex, BMI and smoking status were included in this model**

For their revision, authors should consider all these comments.

Reviewer #2: Event dependent overall survival in the population-based LIFE-Adult-Study
PONE-D-22-10390
Observations
It was interesting reading the article, not just because such studies are rare in developing
countries but also because it has disease diagnosis and risk factors in the data set. The study
demonstrates the role of the disease in the survival time of the adult after controlling the
risk factors. The authors have calculated survival time and cumulative incidence of mortality
from August 2011 until the end of 2019 (line no. 102-104) using the time of the occurrence
of the death out of the cohort of 10,000 adults aged 18-80 years in Leipzig, Germany.
The authors of the paper have attempted and also discussed all the limitations of the cohort
study and elaborated on why there is a difference in the mortality of the members of the
cohort and the general population (Line no. 32-34). I see this as the weakest point of this
paper, as the study findings can not be generalized to the general population of Germany.
This, in no way, undermines the value of the results as it may apply to the specific
population of a particular geographic area. I, however, have the following specific
observations on the present paper:
1. Line no. 72-75- The authors mention that the study subjects are randomly selected
stratified by age and sex. The question that immediately comes to me is if this is so,
then why is there a huge difference in mortality of the cohort and general
population?

We thank the reviewer for this important comment.

We agree that we have bias in our population.

Only about thirty percent of the randomly selected persons participated in the LIFE-Adult baseline examination. Hence the bias.

We have revised and supplemented the text to acknowledge this statement :

249 Since only about thirty percent of the randomly selected persons participated in the LIFE-Adult baseline examination the analesed cohort is not entirely representative.

2. There are two main explanations given: one low participation (33%, Line no. 237-
238) and another one ---selection of study population with higher social status and
healthier lifestyle—---(Line No. 244-245). The question then is, “why such
differences in the population Characteristics”? In my view, it may be that study is
located in a particular geographic location with economically better-off people and a
good lifestyle. The authors must provide explanations for such differences. I did not
find anything on the geographic spread of the study population. The authors need to
include them in the paper. We have revised and supplemented the text to acknowledge this statement (s. below)
3. Line No. 370-387: The last point again is about the natural selection bias. While
trying to explain the absence of some known diseases (Myocardial infarction and
stroke) from the list of the cause of death, authors rely on overlapping effects and
interaction of some causes of death, and therefore the size of contribution gets
reduced. This may be true, but the second explanation needs some evidence. Paper
fails to provide any data for this.
It is hypothesized that those with such severe causes might have died and therefore
have no chance of selection in the study. This seems to me less likely in the
population where all-cause mortality is so low, and the age group for the study is 18-
80 years. If possible, some evidence such as selection bias even from the review may
be included.

We agree. As suggested, we have revised and supplemented the text

255 Some of the non-participants (about 32%) filled a short questionnaire. LIFE-Adult participants reported also less often to have been diagnosed with myocardial infarction , diabetes and stroke as nonparticipants.

259 Consequently, the main health conditions will be underestimated in LIFE-Adult compared to the general Leipzig population. In the multivariate Cox regressionmodels for overall survival we adjusted all diagnoses reported to us with some risk factors as age, gender, BMI, and smoking status.

The overall study contributes to our understanding of survival chances among those who
are diagnosed with some chronic diseases. Therefore, it is worth publishing after some more
explanation, as pointed out in these observations.
Usha Ram

6. PLOS authors have the option to publish the peer review history of their article ([what does this mean?](https://journals.plos.org/plosone/s/editorial-and-peer-review-process#loc-peer-review-history)). If published, this will include your full peer review and any attached files.


If you choose “no”, your identity will remain anonymous but your review may still be made public.


**Do you want your identity to be public for this peer review?** For information about this choice, including consent withdrawal, please see our [Privacy Policy](https://www.plos.org/privacy-policy).

We would like to take this opportunity to thank all editors and reviewers and look forward to hearing from you at your earliest convenience.

With best regards,

Samira Zeynalova and Katja Rillich

Reviewer #1: **Yes:** Ronny Westerman

Reviewer #2: **Yes:** Usha Ram

[NOTE: If reviewer comments were submitted as an attachment file, they will be attached to this email and accessible via the submission site. Please log into your account, locate the manuscript record, and check for the action link "View Attachments". If this link does not appear, there are no attachment files.]

While revising your submission, please upload your figure files to the Preflight Analysis and Conversion Engine (PACE) digital diagnostic tool, <https://pacev2.apexcovantage.com/>. PACE helps ensure that figures meet PLOS requirements. To use PACE, you must first register as a user. Registration is free. Then, login and navigate to the UPLOAD tab, where you will find detailed instructions on how to use the tool. If you encounter any issues or have any questions when using PACE, please email PLOS at [figures@plos.org](mailto:figures@plos.org). Please note that Supporting Information files do not need this step.

*In compliance with data protection regulations, you may request that we remove your personal registration details at any time.* [*(Remove my information/details)*](https://www.editorialmanager.com/pone/login.asp?a=r)*. Please contact the publication office if you have any questions.*
